# Modeling and optimization of argon-based floating helix electrode cold plasma

**DOI:** 10.1038/s41598-025-18837-7

**Published:** 2025-09-29

**Authors:** G. Divya Deepak, Gajanan Anne, Subraya Krishna Bhat

**Affiliations:** Department of Mechanical and Industrial Engineering , Manipal Institute of Technology Manipal Academy of Higher Education , Karnataka 576104 Manipal, India

**Keywords:** Argon, Biomedical devices, Cold plasma, Machine learning, Engineering, Biomedical engineering, Electrical and electronic engineering, Mechanical engineering, Statistics

## Abstract

Cold atmospheric pressure plasma (CAP) technology has vast potential in several technological domains, including biomedical engineering. CAP, also known as non-thermal plasma, is characterized by high-energy electrons while the bulk gas remains near room temperature, allowing for effective plasma treatment without thermal damage—critical for biomedical applications. This paper presents a coupled machine learning and statistical technique-based process modeling and optimization approach for a novel floating-helix electrode-based cold plasma device, operating strictly within the cold plasma regime. An artificial neural network (ANN) model was developed to describe the relationship between the process parameters—supply voltage (SV) and frequency (SF)—and performance parameters—power consumption (P), and jet lengths with and without an end ring (JwER and JwoER). The generality and robustness of the ANN model were confirmed through experimental validation and extrapolative predictions. For multi-response optimization, the composite desirability method was employed. Finally, machine learning models for logistic regression—namely, ANN classifier, K-Nearest Neighbor, and Support Vector Machine—were developed to classify the discharge type within the cold plasma operating range, ensuring its suitability for biomedical applications. The proposed system may hold potential for biomedical use, contingent upon further validation through biological testing.

## Introduction

Atmospheric pressure cold plasma jets based on dielectric barrier discharge (DBD-APCPJs) have been applied in several applications^1–4^. APCPJs have garnered substantial attention in recent years owing to their wide range of industrial and biomedical uses^5–9^. *Cold plasma*, also known as *non-thermal plasma*, refers to a partially ionized gas in which the electron temperature is high (several eV), while the ion and neutral species remain close to room temperature. This temperature disparity allows for plasma generation without significant thermal damage to surrounding materials. Unlike thermal or arc plasmas, CAP operates under near-ambient conditions and is particularly suitable for applications involving heat-sensitive targets, such as biological tissues. In this study, the plasma generated using a floating helix electrode configuration was consistently observed to remain in the *cold plasma regime*, characterized primarily by glow discharges without transition into arc behavior. The modeling and optimization framework developed herein is therefore focused specifically on this regime.

Various plasma jet types relying on different excitation mechanisms—from microwave to DC—and discharge modes such as corona and capacitively coupled discharges have been studied^9–11^; however, DBD-APCPJs have not been thoroughly examined, especially with regard to data-driven modeling and optimization. The performance of the DBD-APCPJ depends on numerous input variables, including the Input supply voltage and frequency and discharge type. This leads to variations in the chemistry of plasma gas, and the identification of reactive species using spectrum information is crucial for a range of applications connected to biomedicine^12–15^.

Research indicates that spectral data responsible for the real-time diagnosis of APCPJ sources have attracted considerable attention. More precisely, for a helium-based DBD, O’Connor et al.^16^ developed a multivariate approach based on principal component analysis to link different optical emission spectrum (OES) peaks to electrical characteristics and electron density. Data analytics is a powerful instrument that can be used to extract information from OES more effectively than traditional physics methodologies. Data-driven approaches have been developed with this in mind. The magnitude of the spectrum datasets makes it possible for data analytics tools and machine learning techniques to effectively identify patterns in such massive datasets^17,18^.

**Fig. 1:**
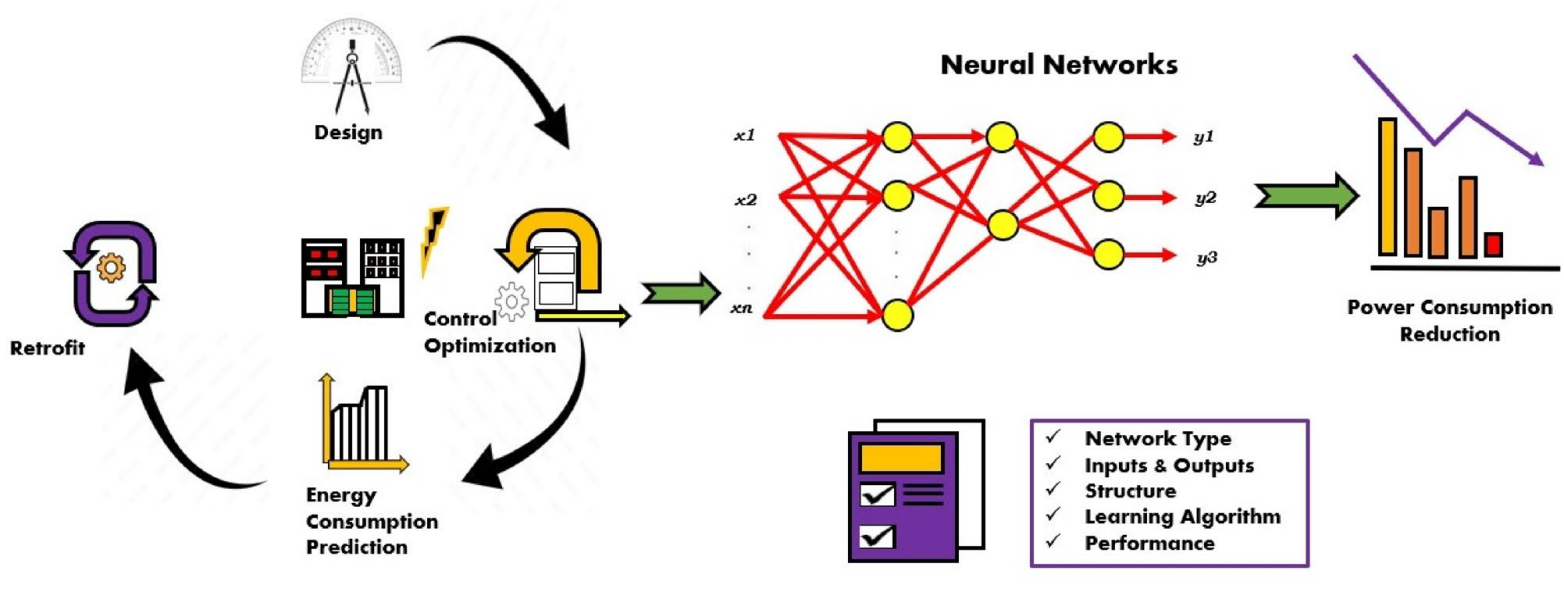
Artificial neural network – applications in process modeling and optimization.

An overview of machine learning (ML) applications for diagnosis, simulation and modeling, and process control of nonequilibrium plasmas was examined in^19^. Chang et al.^20^ studied the plasma discharge current using an artificial neural network (ANN) by employing a deep learning module. They applied a convolutional neural network (CNN) based on a data-driven approach to investigate and extract the current characteristics of the plasma and established that the CNN successfully predicts the work gas and discharge type of plasma discharge. ANN have been employed to predict *the* efficiency of the input parameters of APCPJ in various applications such as food processing, predictive modeling, and CO_2_ splitting^20–24^. The potential of ANN in process modeling and optimization is shown in Fig. 1.

Plasma medicine has extensively used ANNs. Lin et al.^21^ used the ANN technique to estimate the temperature and gas composition using emission spectroscopy; here, the ANN output was used to regulate the power consumption and gas injection rate, thereby optimizing plasma chemistry. Furthermore, in the NO/O_2_/N_2_ system, Wan et al.^25^ anticipated NO conversion by DBD. Their ANN model results demonstrated that the concentration of NO at the inlet, which accounted for 36.22% of the effect, was the primary component that caused the creation of NO_2_. Other factors influencing NO_2_ production were the residence time (26.25%) and discharge power (23.52%).

APCPJ technology comprises several critical operational factors, including power consumption (P), jet length, supply voltage (SV), and supply frequency (SF). From the perspective of cold plasma device development advancement, it is critical to estimate the optimal levels of supply voltage and frequency input parameters to attain the desired levels of power consumption and jet length. In addition, a trustworthy predictive and accurate model can be helpful in generating performance forecasts beyond the range of parameters considered in the experiments, as experimental testing typically has some restrictions with regard to the parameter range.

The goal of this study is to create an ANN model for a new floating helix electrode cold atmospheric pressure plasma (Argon) by training, testing, and validating the model using benchmark experimental data^6^. The robustness of the neural network in representing the physics of the cold plasma device is demonstrated by estimating the performance outside the range of the experimental data utilized for model training. The multi-response optimization process was performed using the desirability function analysis (DFA) technique to predict the ideal input parameter settings for the best performance. Identification of type of plasma discharge of importance to assess the jet’s suitability for biomedical treatment applications. For this purpose, three of the commonly used classical ML classifier models including Support Vector Machine, K-Nearest Neighbors (KNN) and Artificial Neural Network Classifier are implemented to solve a logistic regression (or classification) problem by predicting the nature of discharge zone of the DBD-APCPJ with a floating helix electrode for the previously given combination of inputs. The three models are chosen because of their well-established applicability to several domains of science^15^^,^^21–23^^[,26^. While ML in plasma modeling is established, this study advances: (1) electrode design for stable low-power operation, and (2) interpretable ML-based multi-objective optimization – critical for biomedical adoption where reliability outweighs pure predictive accuracy.

## Experimental setup

This manuscript is based on the experimental work done by^6^ wherein optimized operating range has been found for Ar based cold generation using floating helix and floating end ring configuration. The previous research works done on cold plasma jets for biomedical applications^14,27^ do not employ any floating potential electrode which leads to increased power consumption and also there occurs a transition from glow to arc discharge due to the multipeak discharge phenomenon observed in the I-V characteristics. It is seen that floating helix electrode employed in this research work causes charge accumulation on the dielectric surface around the helix and provides a subsequent discharge path for the plasma jet which results in power consumption only in the range of milli-Watts and longer jet lengths as compared to^14,27^. A floating end ring is used to focus the plasma jet at the outlet of the quartz tube to facilitate the extraction of plasma jet for longer jet lengths as seen in the results. The helix electrode’s pitch in the guiding rail has been experimentally optimized to ensure that the discharge spreads between each consecutive pitch. Moreover, the number of turns has been selected to support the plasma jet’s diffusion outside the quartz tube^6^.

**Fig. 2:**
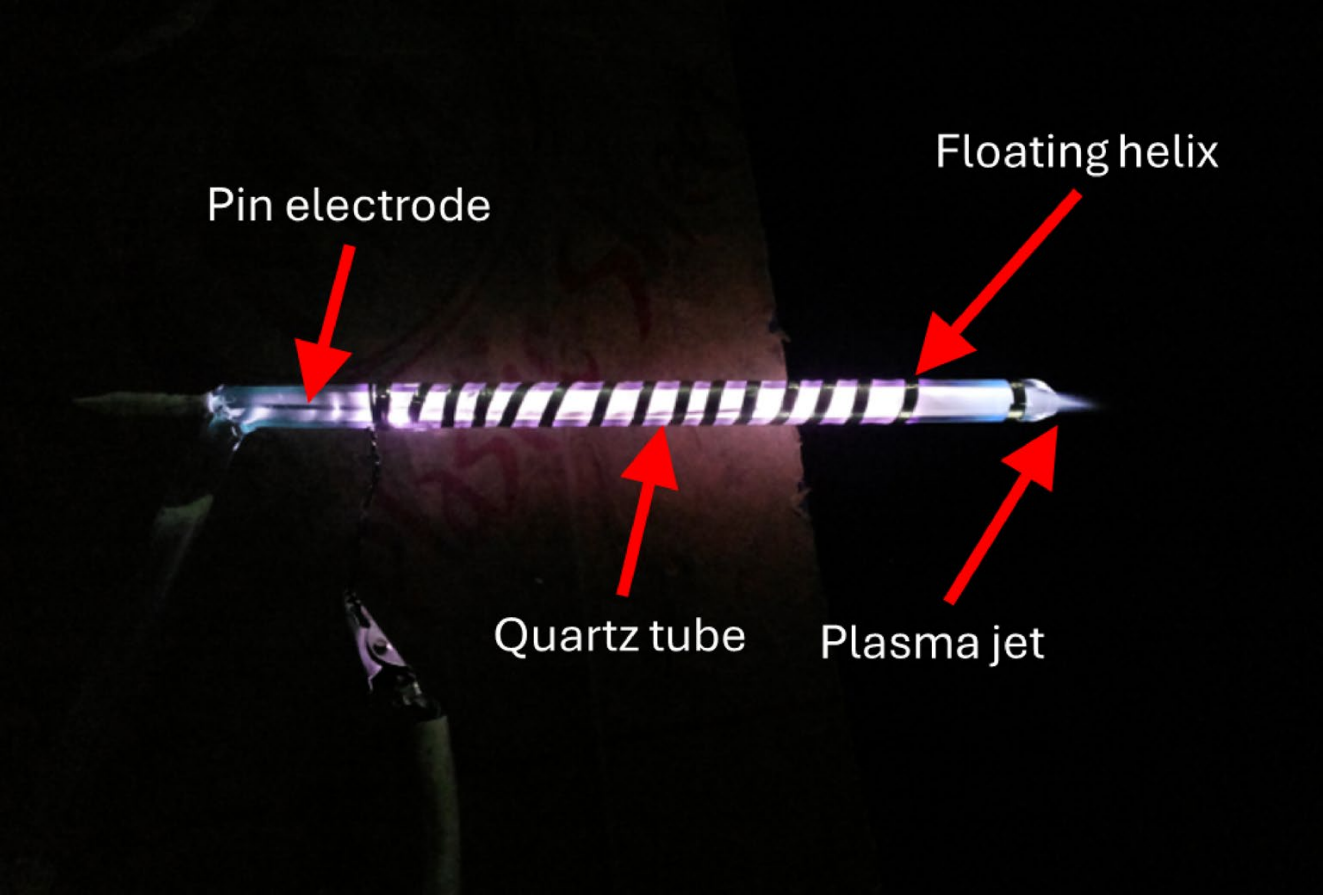
Argon plasma jet obtained using the floating helix based APCPJ.

The geometry of the floating helix electrode (with an end ring)-based APCPJ is illustrated in Fig. 2. The experimental setup consisted of a quartz tube with an inner diameter of 3 mm and 1 mm thickness, which acted as a dielectric barrier between the electrodes. In this geometry, there is a pin electrode with a length of 88 mm and diameter of 1 mm, which acts as the live electrode (cathode) and is sealed hermetically with a quartz tube. The total length of the pin electrodes was 40 mm. An L-shaped glass-blown quartz tube also has an effective plasma discharge length of 123 mm. One end of a quartz tube with a length of 30 mm was utilized for the gas connection, while the other open end was utilized as an outlet for the cold plasma jet. An epoxy silver foil tape of 0.1 mm thickness and width 2 mm was tightly wound around the quartz tube at the gas inlet end and used as the anode. A plasma guiding rail was created using a fourteen-turn helix of epoxy silver foil tape with a pitch of 5 mm. The pitch of the helix electrode was experimentally optimized such that diffused discharge occurred between successive pitches and the number of turns was based on the diffusion of the plasma jet outside the quartz tube.

**Fig. 3:**
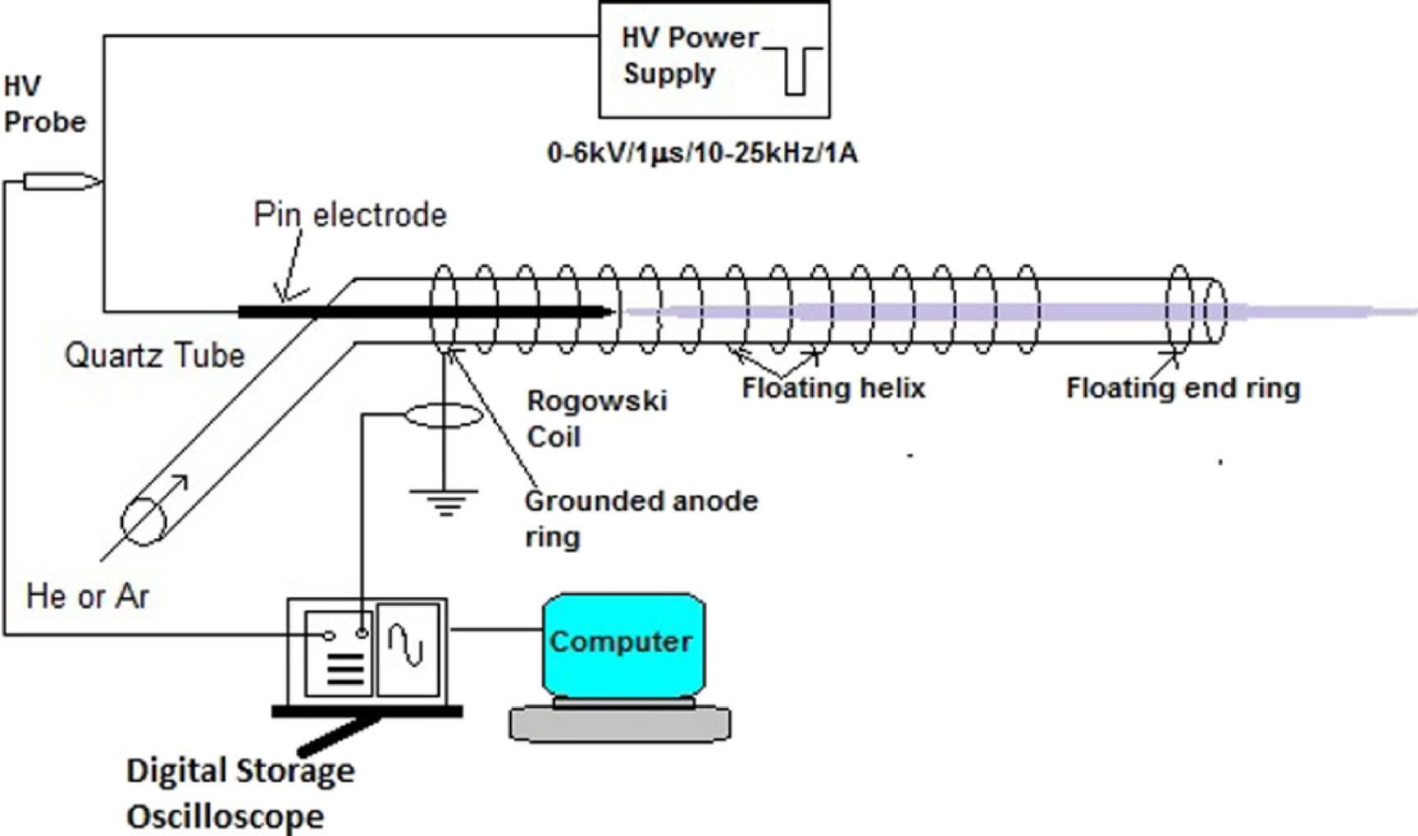
Experimental arrangement of the floating helix electrode configuration with end ring.

The function of the floating helix electrode is to make it easier for charge to build up on the dielectric surface surrounding the helix, thereby providing a channel for the plasma jet to discharge. Nonetheless, the design of the floating helix causes distortions in the plasma jet. Consequently, another end ring electrode (made of silver epoxy) was employed near the exit of the quartz tube outlet 25 mm from the helix to focus the plasma jet (see Fig. 3(a)). In another example, the floating end ring was removed to better understand the effect of the floating end ring on plasma jet extraction for an identical set of input parameters (SV and SF) (see Fig. 3(b)).

As shown in Fig. 3 of the manuscript, the first ring of epoxy silver tape is grounded and serves as the anode, while the floating helix electrode with 14 turns remains unconnected to the anode (not grounded), thus maintaining a floating potential. Additionally, the floating 14-turn helix wound around the quartz tube’s surface prevents arc formation by accumulating charge on the dielectric surface, leading to the generation of multiple micro-discharges across the electrode area^28^. This configuration acts as a guiding rail for the plasma generated, directing it toward the output of the quartz tube, where an end ring helps to direct it, as depicted in Fig. 3.

Jet lengths were measured using calibrated stainless-steel rulers (± 0.1 mm gradation) positioned parallel to the plasma plume, with a demonstrated error margin of ± 0.5 mm accounting for both parallax error (± 0.3 mm) and natural plume fluctuations (± 0.4 mm via high-speed imaging).

The primary function of the floating helix electrode is to provide a geometrical configuration that facilitates charge buildup on the dielectric surface, which in turn aids in the discharge process for the plasma jet. The mechanism behind why charges preferentially accumulate on the dielectric surface can be explained by several key factors:

1. Electric field distribution: The helical structure of the electrode creates a non-uniform electric field, which is concentrated around the dielectric surface. This non-uniform field results in higher electric field intensities near the dielectric, promoting the accumulation of charge on the surface. Such a configuration enables efficient electron trapping, allowing the plasma to form more effectively at the dielectric boundary. Zhu et al.^7^ showed that non-uniform electric field distributions, such as those created by helical electrodes, are more effective at enhancing plasma formation compared to simpler electrode configurations, where the electric field is less concentrated.
2. Capacitive effect: The dielectric material surrounding the electrode acts as a capacitor, where the electrode and dielectric form the two plates, and charge accumulates on the dielectric surface. The presence of the dielectric increases the capacitance of the system, making it easier for charge to build up when a potential is applied. This accumulation is critical for initiating and maintaining the plasma discharge.
3. Surface charge and dielectric properties: Dielectrics, especially those with high permittivity, tend to store surface charge more effectively than conducting materials. This is because the dielectric material prevents the free flow of charge across the surface, allowing it to accumulate in a confined region, which creates an ideal environment for plasma discharge. The floating helix configuration, with its dielectric surface, enhances this effect by facilitating the concentration of charge near the plasma formation region.
4. Charge trapping and localization: The helical design of the electrode also promotes charge localization around the dielectric surface due to the spiraling motion of electrons. The curvature of the helix increases the surface area for charge accumulation, and the electric field is concentrated near these surfaces, ensuring that charge buildup occurs more readily at these sites.

The applied current and voltage were measured using a Rogowski-type current monitor (Make: Pearson Electronics, Model 110; 1 Hz − 20 MHz, 0.1 VA − 1 VA, 20 ns usable rise time) and a high-voltage probe (bandwidth 0–75 MHz, Tektronix P 6015 A) connected to a digital storage oscilloscope (bandwidth 500 MHz, Tektronix DPO 4054). Table 1 lists the experimental results (jet length with and without end ring (JwER and JwoER) in mm, power (P) in Watt) for the different supply voltages (in kV) and supply frequencies (in kHz) for the floating helix-based APCPJ previously presented^6^.

**Table 1:** Experimental results^6^.

Expt. No.	SV (kV)	SF (kHz)	JwER (mm)	JwoER (mm)	*P* (mW)	Discharge type
1	2	10	2	2	0.60	Glow discharge
2	2	15	2	2	0.80	Glow discharge
3	2	20	2	2	1.40	Glow discharge
4	2	25	4	3	1.30	Glow discharge
5	2.5	10	4	3	1.40	Glow discharge
6	2.5	15	4	4	1.48	Glow discharge
7	2.5	20	4	4	1.70	Glow discharge
8	2.5	25	6	5	1.65	Glow discharge
9	3	10	6	6	1.60	Glow discharge
10	3	15	6	6	1.80	Glow discharge
11	3	20	7	6	2.00	Glow discharge
12	3	25	11	9	1.70	Glow discharge
13	3.5	10	12	7	1.80	Glow discharge
14	3.5	15	12	8	2.05	Glow discharge
15	3.5	20	13	8	2.30	Glow discharge
16	3.5	25	17	11	2.10	Glow discharge
17	4	10	18	9	2.00	Glow discharge
18	4	15	19	10	2.30	Glow discharge
19	4	20	19	10	2.50	Glow discharge
20	4	25	22	12	2.70	Glow discharge
21	4.5	10	20	11	2.45	Glow discharge
22	4.5	15	21	11	3.60	Glow discharge
23	4.5	20	22	11	3.85	Glow discharge
24	4.5	25	23	15	3.65	Glow discharge
25	5	10	22	13	3.00	Glow discharge
26	5	15	23	14	5.00	Glow discharge
27	5	20	22	13	5.20	Glow discharge
28	5	25	26	18	5.00	Glow discharge
29	5.5	10	24	13	4.00	Glow discharge
30	5.5	15	24	15	5.90	Glow discharge
31	5.5	20	25	14	6.10	Transition of Glow to Arc
32	5.5	25	29	20	5.90	Glow discharge
33	6	10	25	15	5.00	Glow discharge
34	6	15	26	16	7.10	Transition of Glow to Arc
35	6	20	26	15	7.00	Transition of Glow to Arc
36	6	25	32	22	12.00	Transition of Glow to Arc

## ANN for predictive modeling

The idea behind the ANN, a soft computing method, came from the way a biological species’ nervous system functions. ANN models consist of a set of computational centers called *neurons* wherein the input information is processed through a nonlinear mathematical function (hyperbolic tangent, hyperbolic logarithmic, etc.), arriving at one or more outputs. The nonlinearity introduced in neuronal computations is the key enabling factor that makes ANN an ideal choice for learning and modeling complex processes^29^. In the case of regression problems, ANN results is compared with the experimental results and the mathematical function of the neurons is optimized to minimize this error. This process is called the feedforward backpropagation (FFBP) technique. The feedforward computations taking place in a neuron of the ANN are mathematical function called *activation functions* (or *transfer functions*), such as hyperbolic tangent sigmoid (tansig), logarithmic tangent sigmoid (logsig), and pure linear (purelin).

Typically, the input transferred to a neuron is subjected to a weighted summation along with a numerical term called *bias*^21,23^. This sum passes through the activation function, which introduces nonlinearity to the ANN, and the weights and biases determine whether a neuron is activated. In the simplest arrangement, an ANN comprises one input layer followed by a set of *neurons* according to the problem requirements, which perform computations to establish a suitable mathematical relationship (called the hidden layer), and at the end, The output layer, comprises of the group of output parameters that are to be predicted by the model. The weights (*w*_i_) and biases (*b*_j_) pertaining to each neuron were adjusted to reduce the residual between the predicted and experimental results with the backpropagation process.

In this work, the Levenberg-Marquardt algorithm was used to train a single-layered FFBP ANN model created using MATLAB^®^ (MathWorks, Natick, Massachusetts, USA)^30^. Three response characteristics, namely jet length with and without an end ring (JwER and JwoER), power consumption (P), supply voltage (SV), and supply frequency (SV), are the outputs. The entire experimental dataset, which consists of 36 findings, was split 70:15:15 into training data, testing data, and validation data sets. In MATLAB, the *dividerand* function was used to randomly partition the data for training, testing, and validation. Two approaches were adopted in this study to limit the likelihood of overfitting because of the limited size of the experimental dataset. Firstly, multiple trials of training, testing, and validation were carried out by Changing the number of neurons in the hidden layer from 1 to 10, while altering the number of datasets to prevent predisposition and bias of the data to the chosen group of datasets. Secondly, 5-fold cross-validation was performed by splitting the dataset into 5 subsets, training on one subset and testing on other subsets during each iteration. Furthermore, making new predictions outside the scope of experiments of the control parameters and analyzing the outcomes based on the physics of plasma formation allows one to determine the dependability of the expected data from the trained model.

**Fig. 4:**
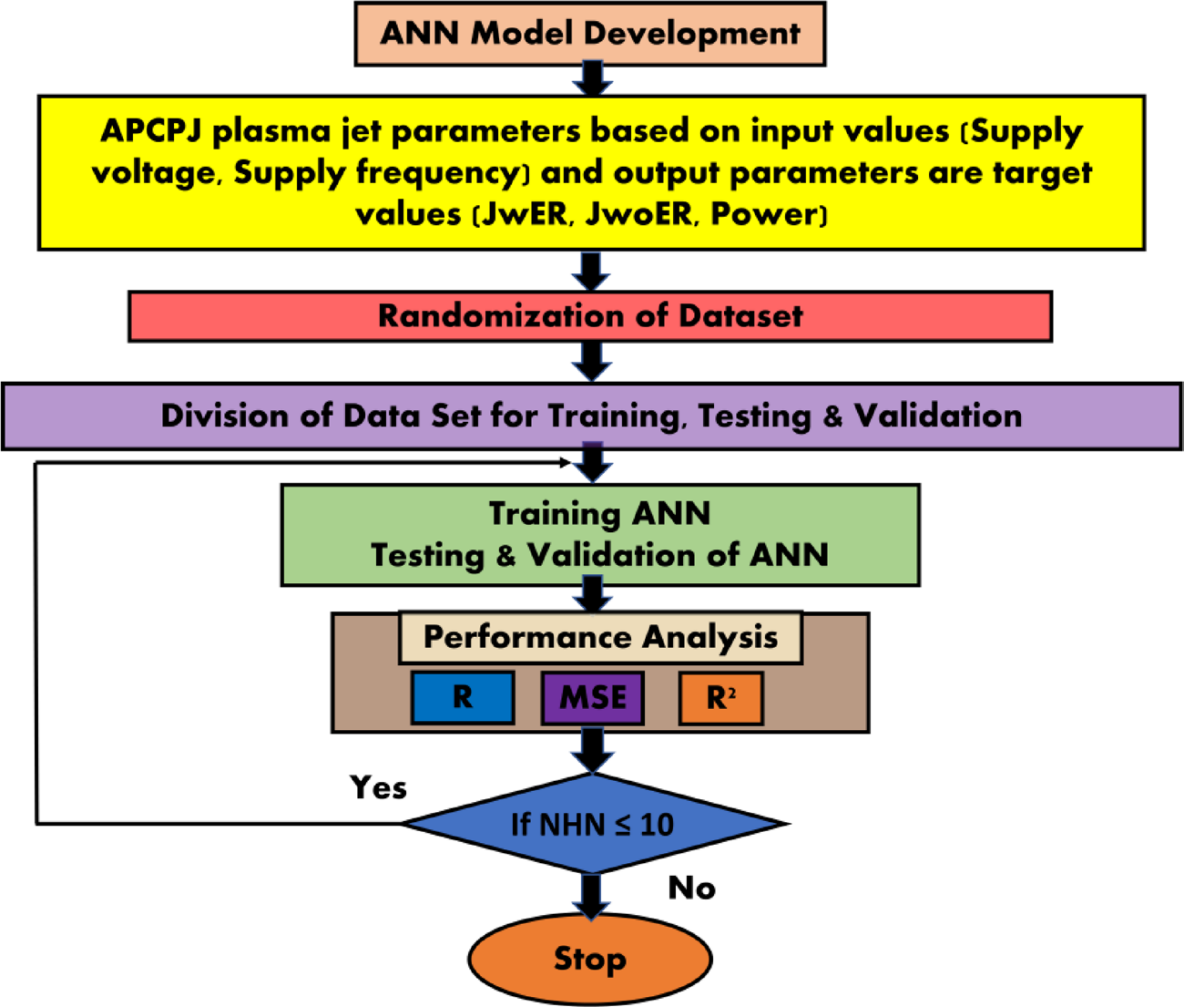
ANN development process.

The coefficients of correlation (R), mean square error (MSE) and determination (R2), were used to gauge the accuracy of the developed model. Figure 4 shows a flowchart of the entire process workflow used to determine the required number of neurons in the ANN.

The governing equations and computations involved in the ANN are summarized herein. The operating parameters (called hyperparameters) in the ANN are the weights ($\:{w}_{k,j}$), biases ($\:{b}_{k}$), and activation function. Initially, the inputs and outputs are normalized between [$\:-1,+1$]. Subsequently, for $\:n$ number of inputs, the outputs obtained at the $\:{k}^{th}$ neuron using the activation function that operates upon the weights and biases can be expressed as^31,32^

1 $${U_k}={b_k}+\sum\limits_{{j=1}}^{n} {{w_{k,j}} \times {I_j}}$$

In the matrix form it can be written as^21,23^

2 $${\left( {\begin{array}{*{20}{c}} {{w_{11}}}& \ldots &{{w_{1n}}} \\ \vdots & \ddots & \vdots \\ {{w_{m1}}}& \cdots &{{w_{mn}}} \end{array}} \right)_{m \times n}}{\left[ \begin{gathered} {I_1} \hfill \\ \vdots \hfill \\ {I_n} \hfill \\ \end{gathered} \right]_{n \times 1}}+{\left[ \begin{gathered} {b_1} \hfill \\ \vdots \hfill \\ {b_m} \hfill \\ \end{gathered} \right]_{m \times 1}}={\left[ \begin{gathered} {U_1} \hfill \\ \vdots \hfill \\ {U_m} \hfill \\ \end{gathered} \right]_{m \times 1}}$$

Finally, the output prediction ‘Y’ in terms of the supplied inputs can be written as^30,32^

3 $$Y={b_{output - layer}}+\sum\limits_{{k=1}}^{m} {L{W_k} \times f\left( {{U_k}} \right)}$$

where $\:{b}_{output-layer}$ and $\:{LW}_{k}$ are the output-layer bias and weight matrix, respectively. The function $\:f\left(x\right)$ used is the tan-sigmoid (*tansig*) activation function given by^30,32^

4 $$f\left( {{U_k}} \right)=\frac{2}{{1 - {e^{ - 2{U_k}}}}} - 1$$

The hidden layer is coupled with the output layer using a linear (*purelin*) function^30,31^. The accuracy of the model predictions was measured by computing the MSE, Pearson’s correlation coefficient (R), and coefficient of determination (R^2^), expressed as^30,32^

5 $$MSE=\frac{1}{n}\sum\limits_{{i=1}}^{n} {{{\left( {{y_i} - {y_{\exp - i}}} \right)}^2}} ,{\text{ }}R=\frac{{\sum\limits_{{i=1}}^{n} {\left( {{y_{\exp - i}} - {{\bar {y}}_{\exp - i}}} \right)\left( {{y_i} - {{\bar {y}}_i}} \right)} }}{{\sqrt {\sum\limits_{{i=1}}^{n} {{{\left( {{y_{\exp - i}} - {{\bar {y}}_{\exp - i}}} \right)}^2}} \sum\limits_{{i=1}}^{n} {{{\left( {{y_i} - {{\bar {y}}_i}} \right)}^2}} } }}{\text{, }}{R^2}=1 - \frac{{\sum\limits_{i}^{n} {{{\left( {{y_i} - {y_{\exp - i}}} \right)}^2}} }}{{\sum\limits_{i}^{n} {{{\left( {{y_i} - {{\bar {y}}_{\exp - i}}} \right)}^2}} }}$$

where $\:{y}_{i}$, $\:{y}_{exp-i}$ are the model predictions and corresponding experimental data, respectively; and $\:{\stackrel{-}{y}}_{i}$ and $\:{\stackrel{-}{y}}_{exp-i}$ indicate their means; and *n* is the size of the dataset for which predictions are being made.

## Optimization of multi-response using desirability function analysis

Desirability Function Analysis (DFA) is a classical multi-criteria decision-making (MCDM) technique widely accepted in the areas of the design of experiments and statistical quality improvement^33^. This allows for the simultaneous optimization of multiple variables involved in a process or system, even those with mutually conflicting behaviors. The DFA method involves converting the overall process response into a single combined quantity called the composite desirability index (CDI), which is obtained as the geometric mean of the desirability value assigned to each response variable (i.e., the desirability index (DI))^34^. Maximization of CDI is the prime objective of DFA, which is expected to optimize the process to achieve the best possible condition for all process response variables. Optimization can be conducted using techniques such as the response surface methodology. The DFA computations begin with the calculation of the desirability index corresponding to the response parameter being maximized or minimized using Eqs. (6) and (7), respectively^34,35^.

6 $${d_i}=\left\{ \begin{gathered} 0{\text{ }}{y_i}<{y_{\hbox{min} }} \hfill \\ {\left( {\frac{{{y_i} - {y_{\hbox{max} }}}}{{{y_{\hbox{max} }} - {y_{\hbox{min} }}}}} \right)^s}{\text{ }}{y_{\hbox{min} }} \leqslant {y_i} \leqslant {y_{\hbox{max} }},s \geqslant 0 \hfill \\ 1{\text{ }}{y_i}>{y_{\hbox{max} }} \hfill \\ \end{gathered} \right.$$

7 $${d_i}=\left\{ \begin{gathered} {\text{1 }}{y_i}<{y_{\hbox{min} }} \hfill \\ {\left( {\frac{{{y_i} - {y_{\hbox{max} }}}}{{{y_{\hbox{min} }} - {y_{\hbox{max} }}}}} \right)^r}{\text{ }}{y_{\hbox{min} }} \leqslant {y_i} \leqslant {y_{\hbox{max} }},r \geqslant 0 \hfill \\ 0{\text{ }}{y_i}>{y_{\hbox{max} }} \hfill \\ \end{gathered} \right.$$

where $\:{y}_{min}$ and $\:{y}_{max}$ are the minimum and maximum responses $\:{y}_{i}$, respectively, $\:s$ and $\:r$ are the weights assigned to a response variable. We assumed equal weightage for each response ($\:s=r=1/3$), based on the following considerations. Maximizing plasma jet length and minimizing power consumption are equally critical to ensuring efficient performance and energy sustainability in cold plasma device applications. Since the generation of a stable and long plasma jet is as important as energy efficiency, it was logical to consider these responses as equally significant. Moreover, at the time of this study, no established industry-specific or application-specific priority metrics were available for these responses. Assigning equal weights also provided a balanced optimization approach without introducing subjective bias. Here, P is the parameter to be minimized, and JwER and JwoER are maximized. CDI is computed as^34,35^

8 $$CD{I_i}={\left( {d_{{i,1}}^{{{w_1}}} \times d_{{i,2}}^{{{w_2}}} \times d_{{i,3}}^{{{w_3}}} \times \ldots \times d_{{i,k}}^{{{w_k}}}} \right)^{1/k}}$$

Here, k represents the number of responses., $\:{d}_{i,k}^{{w}_{k}}$ represent the DI of the $\:{k}^{th}$ response, and $\:{w}_{1}$, $\:{w}_{2}$, $\:{w}_{3}$,…, are the corresponding weights such that their summation becomes 1. We assumed equal weights for all responses. To determine the optimal set of control settings that maximizes CDI, the mean factorial effect was examined^35^. The optimal combination of the control parameter settings for the multiple-response problem is indicated by the maximum CDI.

## ML models for discharge type classification

The application of the cold plasma technique in biomedical applications necessitates the generation of plasma at room temperature to avoid thermal damage to biological tissues. Physically, it is necessary for the plasma to operate in the glow discharge region and strictly stay away from the arc discharge zone. A certain electrode shape and set of operational parameters were essential to retain the discharge in the glow regime at atmospheric conditions pressure. The region of discharge (glow discharge and glow-to-arc discharge transition) was predicted using logistic regression models. First, based on the experimental results, the discharge classes were labeled (see Table 1). The ML models were then trained using experimental data including all the experimental parameters including SV, SF, JwER, JwoER and P as inputs, to forecast the type of discharge. The training and testing datasets were split in a 90:10 ratio.

This study evaluates three different classification techniques: Support Vector Machine (SVM), K-Nearest Neighbors (KNN), and Artificial Neural Network Classifier (ANNC). KNN is a simple, instance-based learning algorithm that performs classification based on feature similarity. It is non-parametric, making no prior assumptions about the data distribution, thus serving as a baseline for performance evaluation in scenarios where relationships between features may not be explicitly known^26^. SVM excels at finding the optimal hyperplane for class separation, even in high-dimensional spaces. It is effective with complex datasets due to its ability to handle non-linear boundaries using kernel functions. SVM also has strong theoretical foundations, making it a standard benchmark for classification tasks^36^. ANNC is a powerful model capable of learning complex and non-linear patterns through multiple layers of interconnected neurons. Its adaptive learning nature makes it suitable for capturing intricate feature representations, complementing the simpler models in this study. Stratified 5-fold cross-validation was implemented to reduce overfitting and provide a more reliable estimate of performance. Together, these three models provide a balanced comparison of simple to complex data-handling capabilities, ensuring robust evaluation of classification performance across diverse feature spaces.

### K-nearest neighbors

The K-nearest neighbor (KNN) technique, a fundamental supervised machine learning algorithm, was developed to handle tasks related to both regression and classification. However, it has been frequently applied for classification purposes. With KNN, a number of data instances of K-neighbors are chosen using the nearest distance measure (e.g., Correlation, Euclidean, or Mahalanobis)^37^. The new data point was then assigned to the category with the greatest number of neighbors after the number of data instances in each category was calculated. One major advantage of the KNN classification is its stability and efficiency when handling large training datasets^38^. Here, the distance metric and number of neighbors are the hyperparameters considered for optimization.

### Support vector machine

SVM, which was created in the 1960 s, started to gain popularity in the 1990 s, while it underwent substantial improvements^39^. It is currently regarded as an effective ML technique with superior accuracy and low computational power requirements. SVM is frequently employed for classification goals and can also be used for regression. By looking for the best hyperplane or decision boundary (in an N-dimensional space, where N is the number of characteristics or input factors), the SVM attempts to categorize the dataset into classes or categories^40^. The decision hyperplane dimensions depend on the number of control factors. For instance, if two input features exist, the hyperplane becomes a line, and its dimensions increase with more features. Here, the hyperparameters considered for optimization are the type of kernel function: Gaussian, Linear, Quadratic and Cubic.

### ANN classifier

Section 3 describes methods and computing procedure used in the ANN regression approach. When it comes to classification problems, the only distinction is that unlike regression problems where the output layer predicts a numerical output prediction, an ANN classifier (ANNC) predicts the class of the output response for a particular set of input parameters in classification problems by computing a classification score^41^. During the training phase, we adjusted the Activation function (among Sigmoid, ReLU, & Tanh) number of fully linked layers (we considered one to three layers), and number of neurons in each layer (we considered 1 to 300 neurons).

### Classifier interpretability analysis using local interpretable model-agnostic explanations (LIME) method

Local Interpretable Model-Agnostic Explanations (LIME) is a method intended to describe the predictions of complex machine learning models by approximating them with interpretable models locally around a specific prediction^42^. This method helps in understanding the influence of input features on the model’s prediction for individual instances. LIME generates explanations by creating a local surrogate model, usually a linear regression or decision tree, which approximates the complex model $\:f$ near the prediction of interest $\:x$. Here we used a linear regression model for this purpose. The method involves generation of perturbed samples $x^{\prime}$ from the vicinity of $\:x$, at which predictions are obtained from the original model as $f\left( {x^{\prime}} \right)$. A location-aware weighting function $\pi \left( {x,x^{\prime}} \right)$ is applied to ensure that samples closer to xxx are given more importance. Typically, an exponential kernel is used:

9 $$\pi \left( {x,x^{\prime}} \right) = \exp \left( { - \frac{{d\left( {x,x^{\prime}} \right)^{2} }}{{\sigma ^{2} }}} \right)$$

where $d\left( {x,x^{\prime}} \right)$ is the distance between the original and perturbed points, and $\:\sigma\:$ controls the spread. The surrogate model $\:g$ minimizes the loss function:

10 $$Loss\left( {f,g,\pi } \right) = \sum\limits_{{x^{\prime}}} {\pi \left( {x,x^{\prime}} \right)\left( {f\left( {x^{\prime}} \right) - g\left( {x^{\prime}} \right)} \right)^{2} } + \lambda \Omega \left( g \right)$$

where $\Omega \left( g \right)$ is a complexity penalty ensuring interpretability. The surrogate model’s learned coefficients are extracted, which represent the contribution of each feature toward the prediction, providing an interpretable explanation for the original model’s decision. A positive coefficient suggests that increasing the feature value raises the prediction score, while a negative coefficient indicates a lowering effect. These coefficients help explain how different features influence the predictions in an interpretable way.

## Results and discussion

### ANN predictive accuracy

**Fig. 5:**
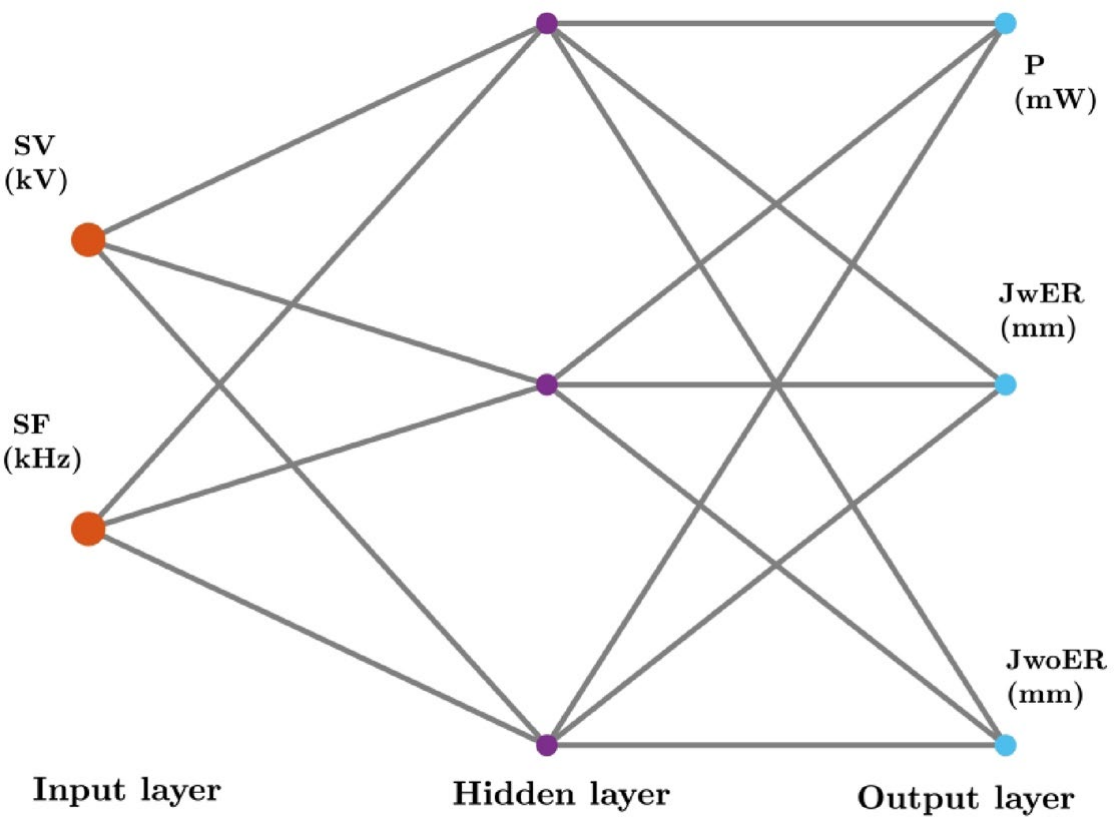
‘2-3-3’ ANN topology.

**Fig. 6:**
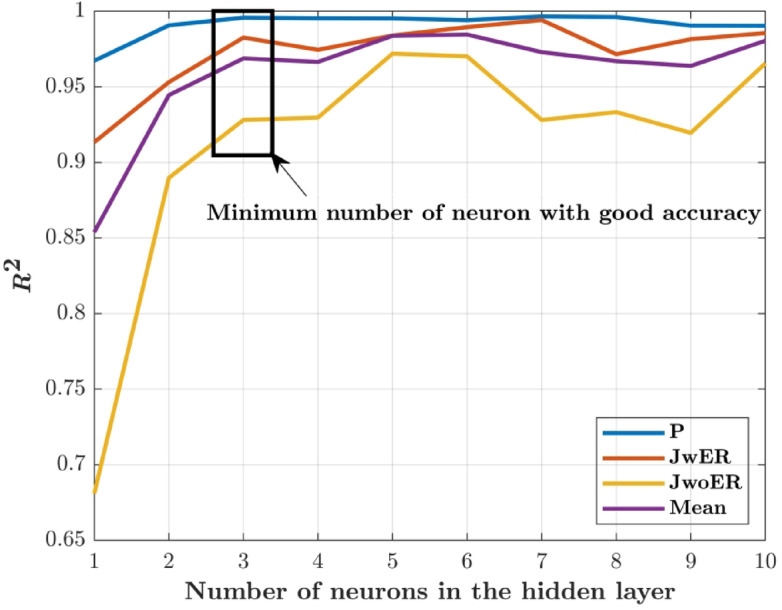
Identification of optimum number of neurons.

**Fig. 7:**
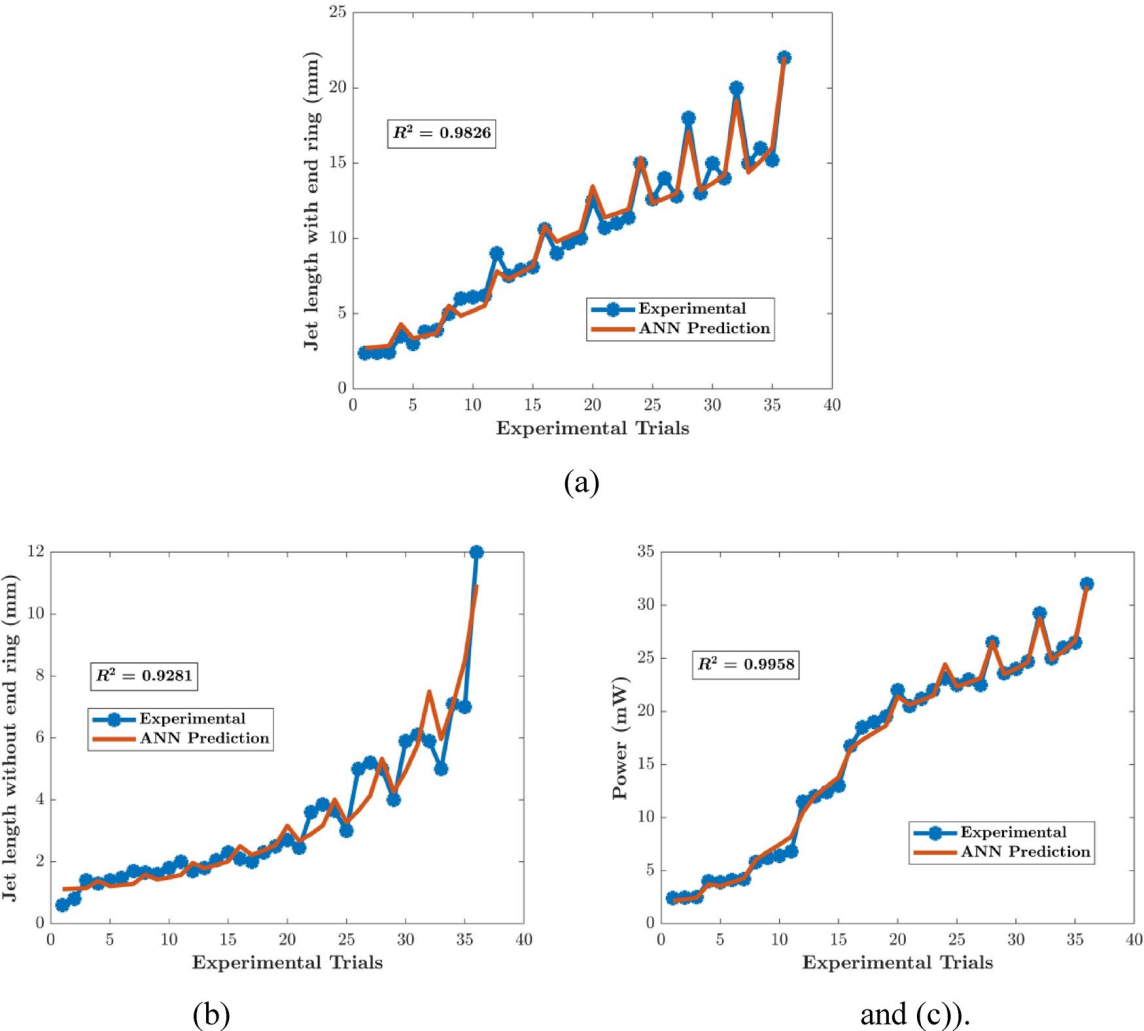
ANN predictions compared with experimental results: (**a**) JwER, (**b**) JwoER and (**c**) P.

**Fig. 8:**
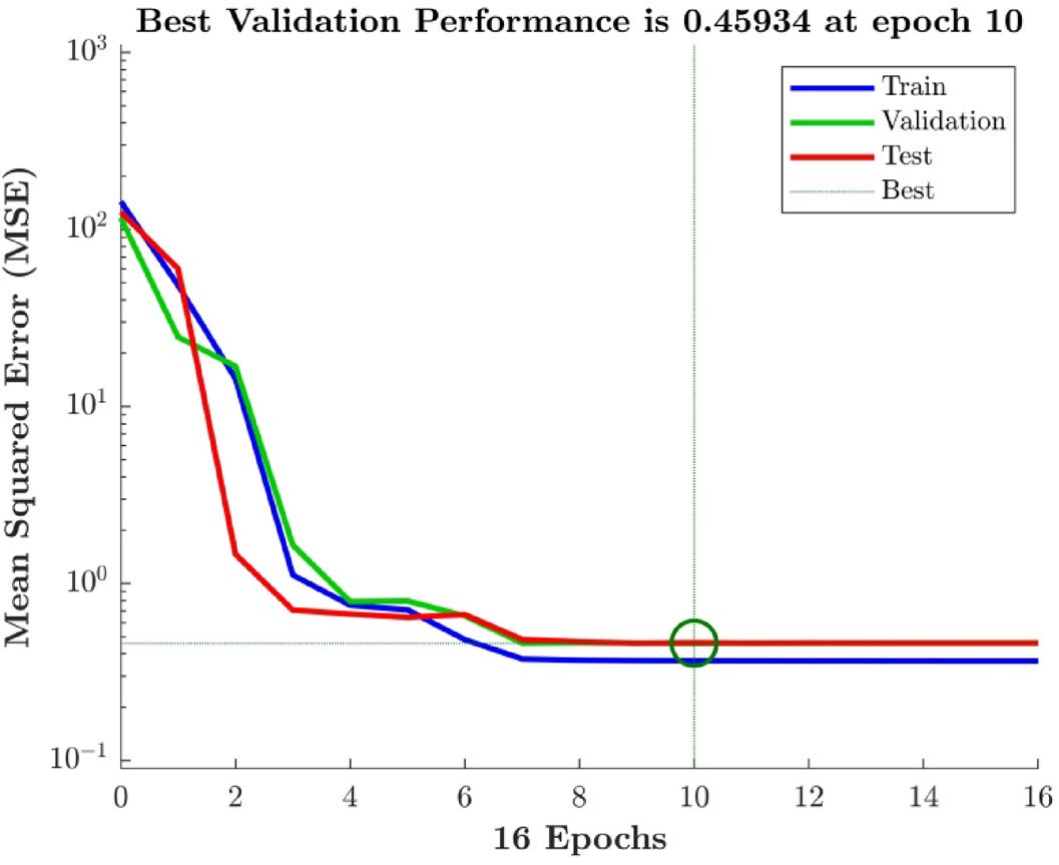
MSE variation during backpropagation.

The neuron number in the hidden layer of the FFBP ANN model was varied by trial and error. With R^2^ > 0.92 for all parameters, the single-layer structure with three neurons (Fig. 5) exhibited the highest descriptive accuracy. Figure 6 shows the fluctuation in R^2^ with respect to the number of neurons in the buried layer. The optimal arrangement to prevent the propensity of overfitting was determined by selecting the least number of neurons with a decent accuracy, even though a larger number of neurons yielded better accuracy (Fig. 7). The ANN predictions for the experimental data listed in Table 2. The MSE fluctuations and correlation coefficients (R) across the training, validation, and testing stages are shown in Figs. 8 and 10, respectively.

**Fig. 9:**
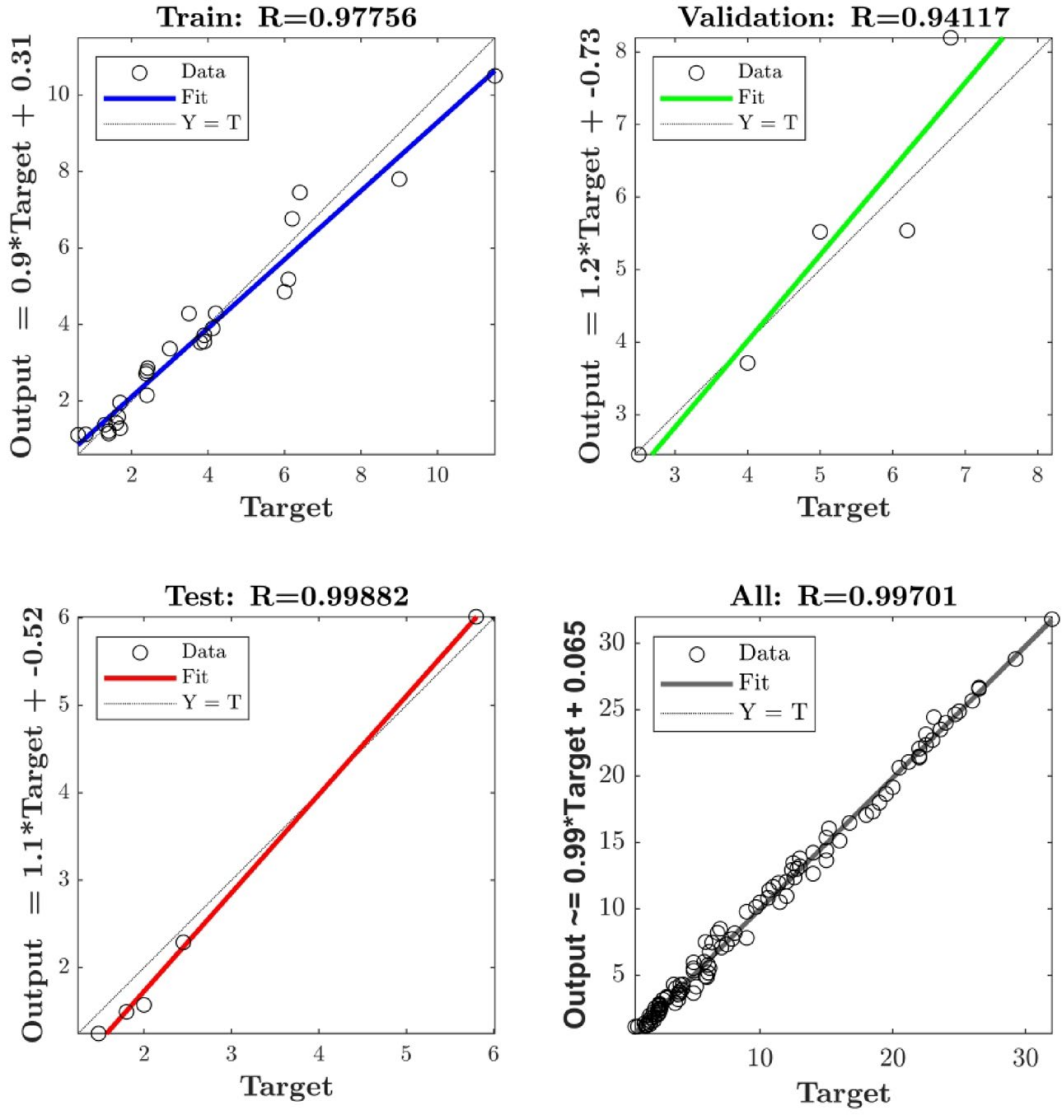
Pearson’s correlation coefficient (R) plots for the optimized ANN.

**Table 2:** ANN predictions (2 kV ≤ SV ≤ 10 kV and 10 kHz ≤ SF ≤ 25 kHz).

Expt. No.	SV (kV)	SF (kHz)	JwER (mm)	JwoER (mm)	*P* (mW)
1	2	10	2.15	2.71	0.56
2	2	15	2.29	2.78	0.75
3	2	20	2.45	2.86	1.25
4	2	25	3.71	4.29	1.37
5	2.5	10	3.55	3.37	1.31
6	2.5	15	3.89	3.53	1.34
7	2.5	20	4.29	3.72	1.59
8	2.5	25	6.01	5.52	1.58
9	3	10	6.77	4.85	1.42
10	3	15	7.45	5.18	1.69
11	3	20	8.19	5.54	1.87
12	3	25	10.50	7.80	1.66
13	3.5	10	12.04	7.31	1.79
14	3.5	15	12.92	7.73	1.99
15	3.5	20	13.80	8.16	2.12
16	3.5	25	16.47	10.82	2.21
17	4	10	17.32	9.78	2.22
18	4	15	17.99	10.13	2.36
19	4	20	18.65	10.48	2.54
20	4	25	21.39	13.46	2.66
21	4.5	10	20.63	11.39	2.50
22	4.5	15	21.05	11.66	2.89
23	4.5	20	21.49	11.95	3.60
24	4.5	25	24.44	15.36	3.55
25	5	10	22.34	12.36	3.18
26	5	15	22.71	12.65	4.85
27	5	20	23.14	13.01	5.14
28	5	25	26.57	17.06	5.02
29	5.5	10	23.51	13.21	3.90
30	5.5	15	24.01	13.65	5.82
31	5.5	20	24.64	14.22	5.97
32	5.5	25	28.81	19.15	5.79
33	6	10	24.87	14.39	5.17
34	6	15	25.67	15.12	7.09
35	6	20	26.67	16.05	6.99
36	6	25	31.81	22.06	11.85
37	6.5	10	26.91	16.26	8.79
38	6.5	15	28.16	17.41	10.57
39	6.5	20	29.64	18.79	12.67
40	6.5	25	35.89	26.03	15.97
41	7	10	29.96	19.08	13.13
42	7	15	31.70	20.69	15.61
43	7	20	33.63	22.49	18.35
44	7	25	40.91	30.89	22.27
45	7.5	10	34.02	22.84	18.92
46	7.5	15	36.07	24.75	21.85
47	7.5	20	38.17	26.69	24.82
48	7.5	25	46.17	36.01	28.85
49	8	10	38.56	27.06	25.39
50	8	15	40.56	28.92	28.25
51	8	20	42.43	30.65	30.89
52	8	25	50.86	40.62	34.55
53	8.5	10	42.75	30.95	31.38
54	8.5	15	44.36	32.44	33.67
55	8.5	20	45.75	33.74	35.63
56	8.5	25	54.54	44.31	38.74
57	9	10	45.98	33.94	35.98
58	9	15	47.08	34.97	37.55
59	9	20	47.99	35.83	38.84
60	9	25	47.26	37.12	41.49
61	9.5	10	47.13	35.94	39.05
62	9.5	15	47.81	36.57	40.03
63	9.5	20	47.37	37.10	40.79
64	9.5	25	49.26	37.27	43.20
65	10	10	42.43	34.15	40.91
66	10	15	43.83	33.52	41.48
67	10	20	42.16	32.83	41.91
68	10	25	41.81	31.01	41.24

The predictive capacity of the ANN model was examined by projecting the performance outside the experimental range (Expt. No. 37–68 in Table 2). A linear relationship was observed between the supply frequency of up to 20 kHz and the power utilized for supply voltages between 2 and 5 kV. Nevertheless, there was a decrease in the power used when the supply frequency was increased to 25 kHz. This is caused by *memory charges*, which can also lead to ionization of argon atoms and a decrease in power. During the first half of the applied voltage phase in DBDs, the remaining species allow for subsequent discharge processes by initiating the creation of seeding electrons and elevating the initial field. This phenomenon is often called the “*memory effect*”^43^. Higher voltages, such as 6 kV and 10–15 kHz, result in an increase in power consumption because the input energy is wasted as heat in the dielectrics and does not help to create plasma.

Additionally, as shown in Table 2, the plasma jet lengths with end rings are longer than the jet lengths without end rings for the majority of supply voltage and frequency instances (2.5 kV − 6 kV, 10–25 kHz). The plasma jet was focused and extracted via the quartz tube exit with the assistance of a floating end ring. The increased jet lengths in Table 2 clearly demonstrate the significant rise in power consumption for SV and SF (6.5 kV − 8.5 kV, 10–25 kHz), where the input power is used for plasma formation. The power usage varies only slightly (2 mW − 6 mW) at even higher voltages of 9 kV − 10 kV (10–25 kHz), as the supply power is lost as thermal dissipation and lost in the dielectric material as seen Table-2.

In contrast to the lower voltage range (8.5 kV, 25 kHz), where JwER = 54.54 mm and JwoER = 44.31 mm, it is important to highlight that the jet length considerably reduced at higher voltages (9 kV, 10 kHz), where JwER = 45.97 mm and JwoER = 33.94 mm. This advocates that up to SV = 8.5 kV, SF = 25 kHz, the power supplied was efficiently used for generation of plasma, as evidenced by the power consumed (38.74 mW at 6.5 kV, 25 kHz). Moreover, at higher voltages (SV = 9 kV − 10 kV, SF = 10–25 kHz), most of the supplied power is lost due to thermal dissipation, consequently, leads to heating of quartz tube instead of utilized for plasma formation. This leads to a decrease in plasma jet lengths at 9 kV, 10 kHz (JLwER = 45.97 mm, JwER = 33.94 mm) and 10 kV, 25 kHz (JwER = 41.81 mm, JwoER = 31.01 mm) proves this point. For every type of working gas medium and DBD-APCPJ arrangement, there is, thus, an optimal operating zone.

### Multi-response optimization

Table 3 presents the DI values for the experimental trials and CDI to optimize the parameters for multiple responses. The CDI was ranked in descending order to assess the best combination of operating parameters to achieve the best performance of the floating helix electrode-based DBD-APCPJ. It was found that SV = 5.5 kV and SF = 25 kHz (corresponding to Expt. No. 32) exhibited the best performance. The factorial effects on CDI were assessed using main effect analysis (Fig. 11). The mean of means of CDI is maximized for 5 kV ≤ SV ≤ 5.5 kV with a marginal effect of SF, which aligns with the results from DFA. Furthermore, Expt. No. 32, with SV = 5.5 kV and SF = 25 kHz achieved glow discharge (see Table 1), meeting the necessary conditions for biomedical applications.

The frequency (25 kHz) falls within the biologically safe range (1–30 kHz) to minimize cellular stress, and the low power consumption (5.90 mW) further reduces thermal risks. The resultant jet lengths (JwER = 29.25 mm) are practical for targeted treatments, as evidenced by prior studies^44,45^. While DFA guided parameter selection, their alignment with plasma physics and biomedical standards underscores their suitability for future in vivo validation.

**Table 3:** Ranking of the process parameters using DFA.

SV (kV)	SF (kHz)	Individual desirability ($\:{d}_{i}$)			CDI		Rank	
		JwER	JwoER	*P*				
2	10	0.00	0.00	1.00	0.00		36	
2.5	10	0.37	0.32	0.98	0.49		32	
3	10	0.50	0.57	0.97	0.65		27	
3.5	10	0.69	0.64	0.96	0.75		24	
4	10	0.82	0.62	0.96	0.78		20	
4.5	10	0.85	0.70	0.94	0.82		19	
5	10	0.88	0.80	0.92	0.87		5	
5.5	10	0.89	0.82	0.89	0.87		7	
6	10	0.91	0.86	0.85	0.88		4	
2	15	0.12	0.10	0.99	0.23		34	
2.5	15	0.39	0.42	0.97	0.54		30	
3	15	0.51	0.57	0.96	0.66		26	
3.5	15	0.70	0.66	0.96	0.76		23	
4	15	0.82	0.72	0.95	0.83		17	
4.5	15	0.86	0.76	0.90	0.84		15	
5	15	0.89	0.84	0.85	0.86		8	
5.5	15	0.90	0.86	0.81	0.86		9	
6	15	0.93	0.89	0.75	0.85		10	
2	20	0.15	0.13	0.98	0.26		33	
2.5	20	0.39	0.43	0.97	0.55		29	
3	20	0.53	0.58	0.96	0.66		25	
3.5	20	0.71	0.66	0.95	0.76		22	
4	20	0.83	0.73	0.94	0.83		16	
4.5	20	0.87	0.77	0.89	0.84		13	
5	20	0.88	0.81	0.84	0.84		14	
5.5	20	0.91	0.84	0.80	0.85		12	
6	20	0.93	0.87	0.76	0.85		11	
2	25	0.38	0.39	0.98	0.52		31	
2.5	25	0.49	0.51	0.97	0.62		28	
3	25	0.67	0.70	0.97	0.77		21	
3.5	25	0.79	0.75	0.95	0.82		18	
4	25	0.87	0.80	0.93	0.87		6	
4.5	25	0.89	0.86	0.90	0.88		3	
5	25	0.93	0.93	0.85	0.90		2	
5.5	25	0.97	0.96	0.81	0.91		1*	
6	25	1.00	1.00	0.00	0.00		36	

*Operating settings of the floating-helix electrode-based DBD-APCPJ for maximum CDI

**Fig. 10:**
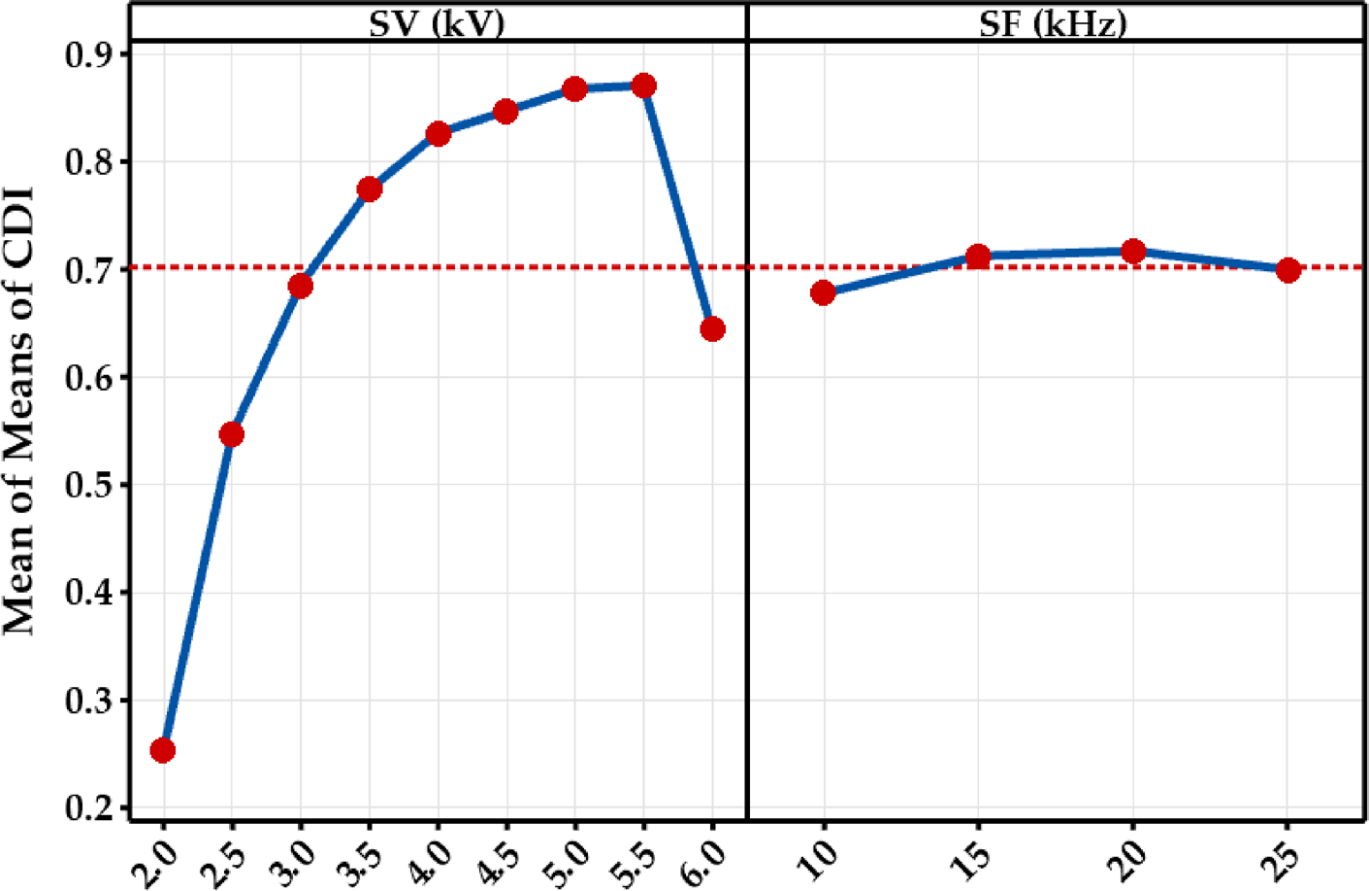
Main effect plot of CDI.

### Regression model sensitivity

The The responsiveness of the optimized ANN model (with the “2-3-3” topology) was evaluated by eliminating one input at a time. The accuracy of the prediction remained almost constant at *R* = 0.98 when SF was not supplied to the model but significantly decreased to *R* = 0.25 when SV was not supplied to the model (Fig. 12). This suggests that SV has a significant effect on the output parameters, whereas SF has a negligible effect. This link is confirmed by the Pearson correlation coefficient (R) between the input and output variables. (Fig. 13), where SF has an *R* < 0.26 and SV has an *R* > 0.85. This provides statistical support for the idea that SV has a greater impact on the reaction than SF does.

**Fig. 11:**
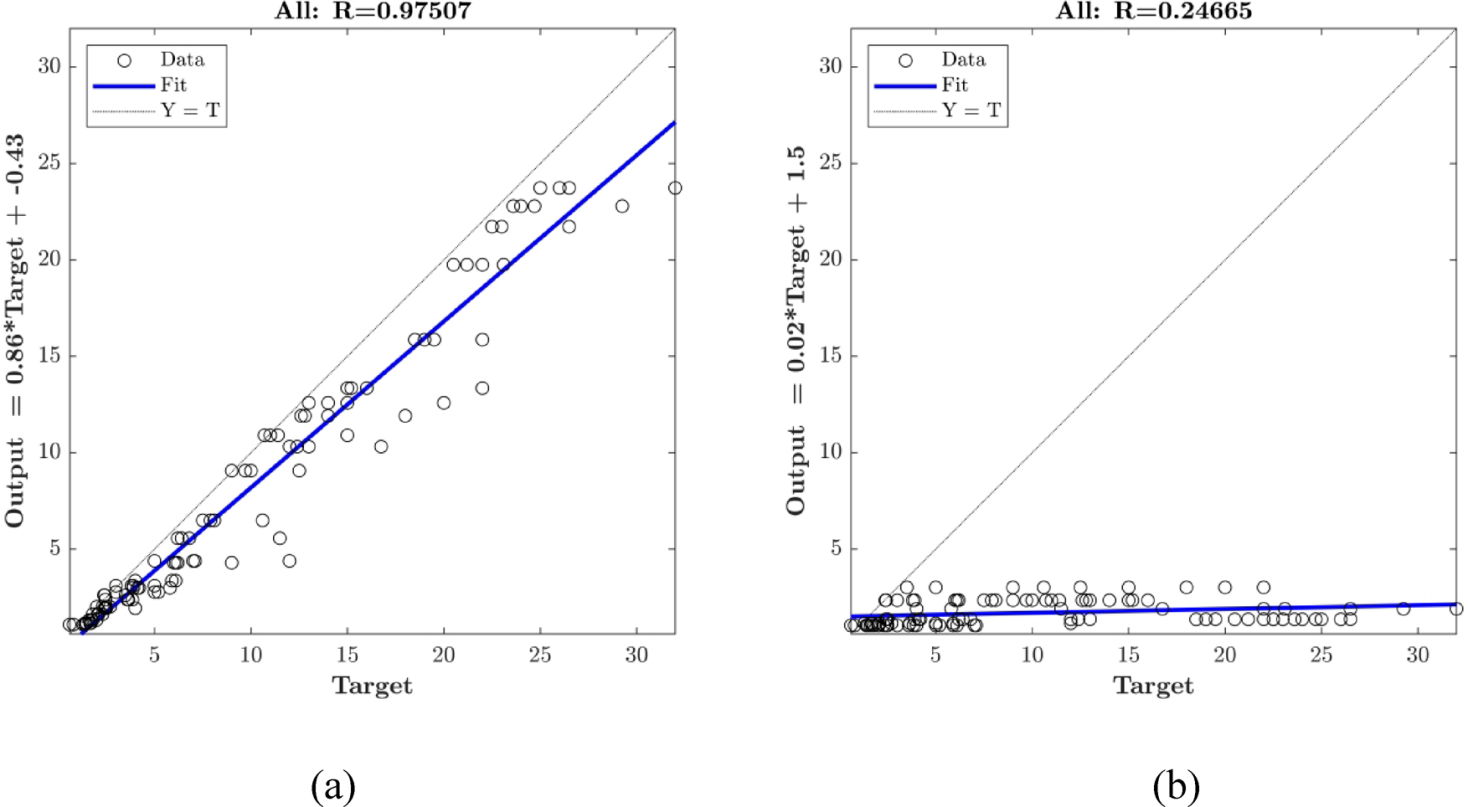
ANN model sensitivity on (**a**) SF and (**b**) SV.

**Fig. 12:**
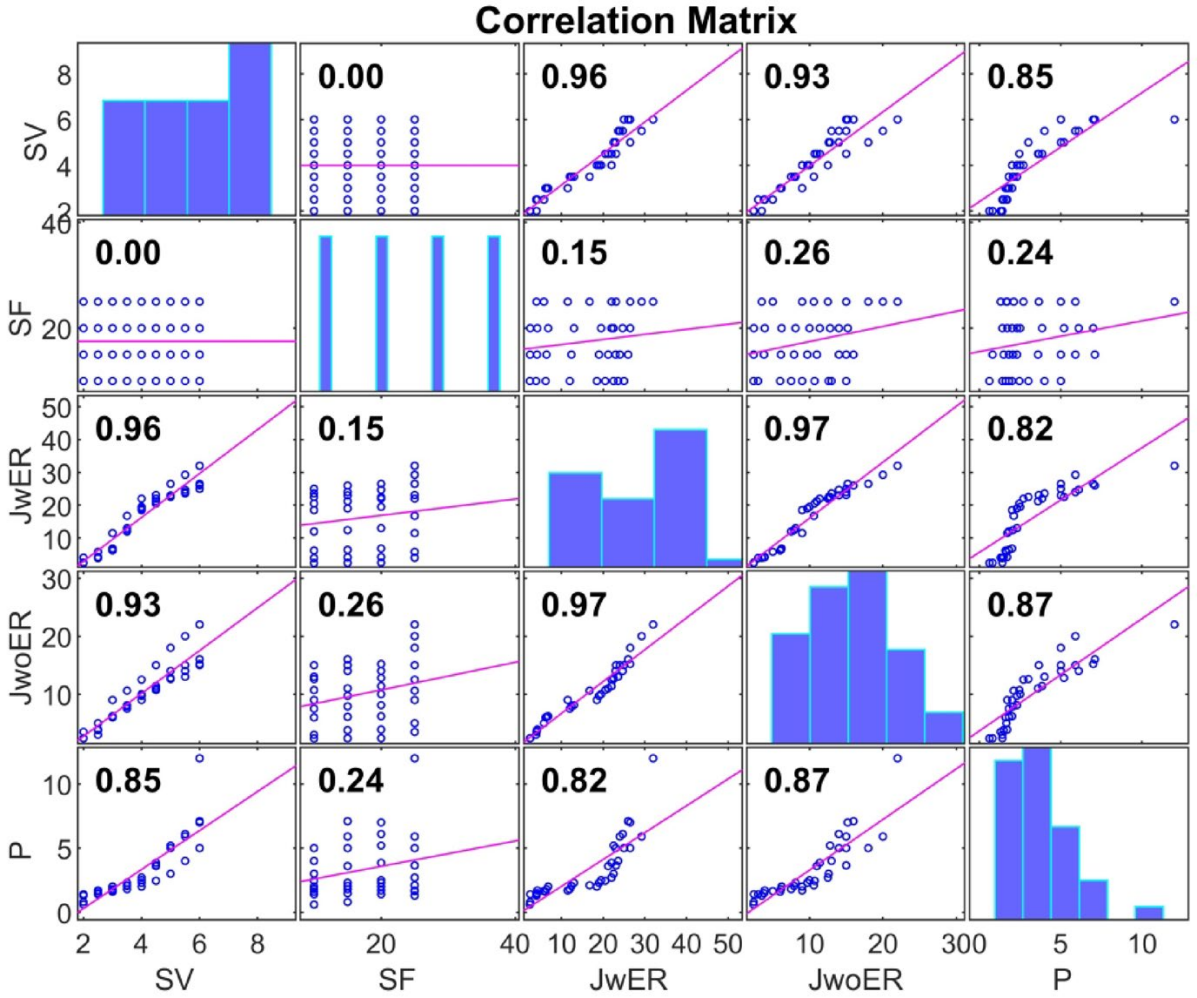
Pearson’s correlation coefficient matrix for experimental results.

### Discharge type prediction performance

**Fig. 13:**
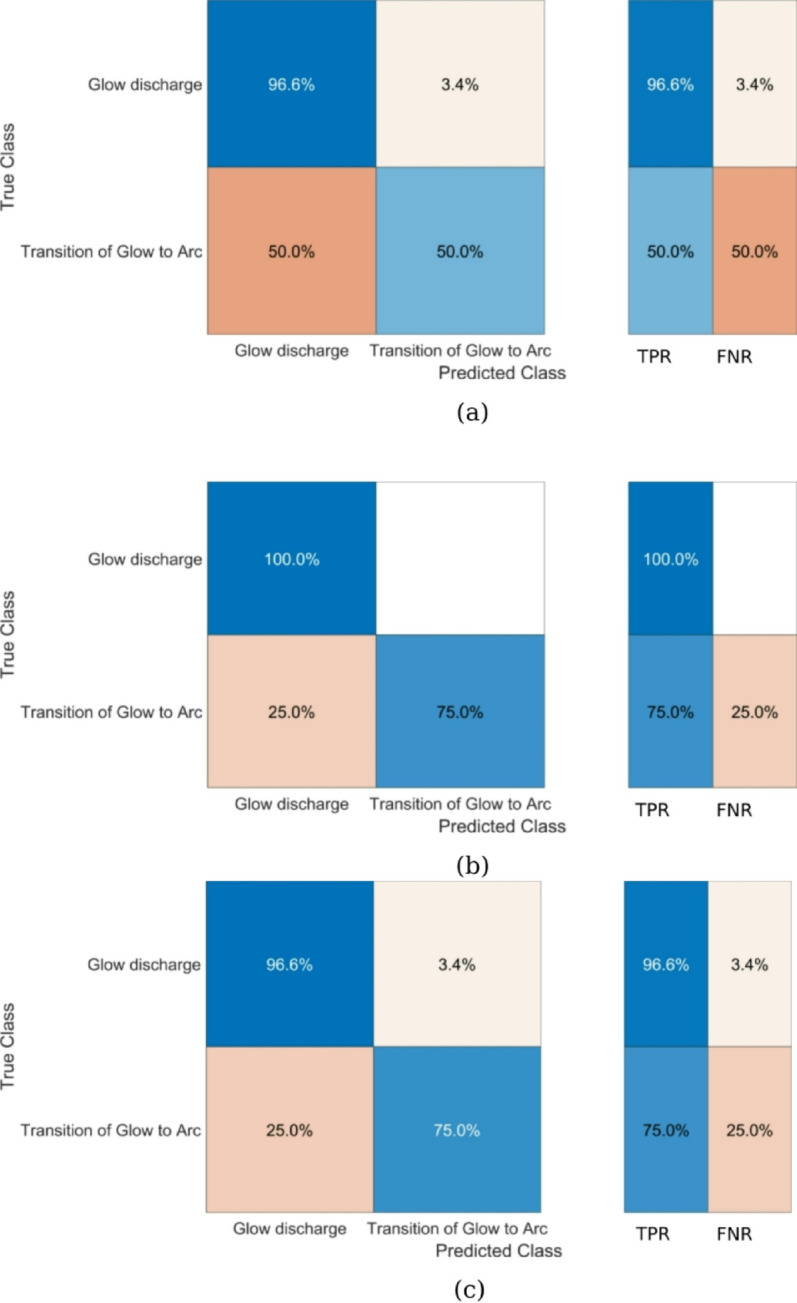
Confusion matrix for (**a**) KNN, (**b**) SVM and (**c**) ANNC models.

**Fig. 14:**
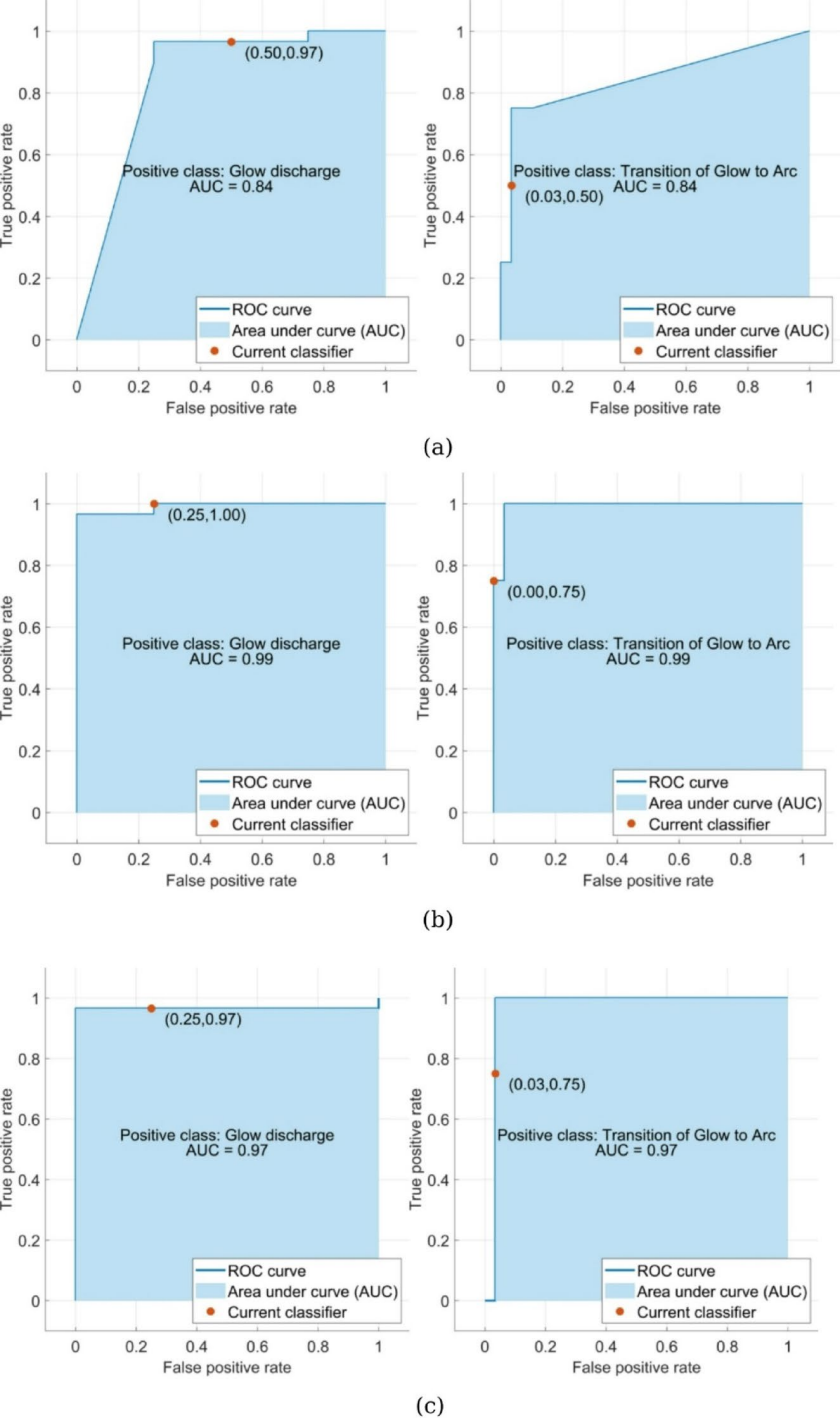
ROC for transition from glow to arc discharge (right column) & glow discharge (left column) with respect to (**a**) KNN, (**b**) SVM and (**c**) ANNC models.

The KNN model (K = 3, correlation distance metric) achieved 96.6% accuracy for arc discharge but only 50% accuracy for transition phases, with an overall AUC of 0.84 ± 0.03 (95% CI). This performance gap reflects the model’s sensitivity to class imbalance in our dataset. The confusion matrix shows particular weakness in identifying transition states between glow and arc discharges. (Fig. 13). In contrast, the SVM model with quadratic kernel function demonstrated superior performance, achieving perfect classification (100% accuracy) for glow discharge and 75% accuracy for transition states. Its exceptional overall AUC of 0.99 ± 0.01 (Fig. 14) for glow discharge classification makes it the most reliable predictor among the tested models. The single-layer ANN Classifier (25 neurons, ReLU activation) showed strong but slightly inferior performance to SVM, with 96.6% accuracy for arc discharge, 75% accuracy for transitions, and an AUC of 0.98 ± 0.01 (Fig. 14).

**Fig. 15:**
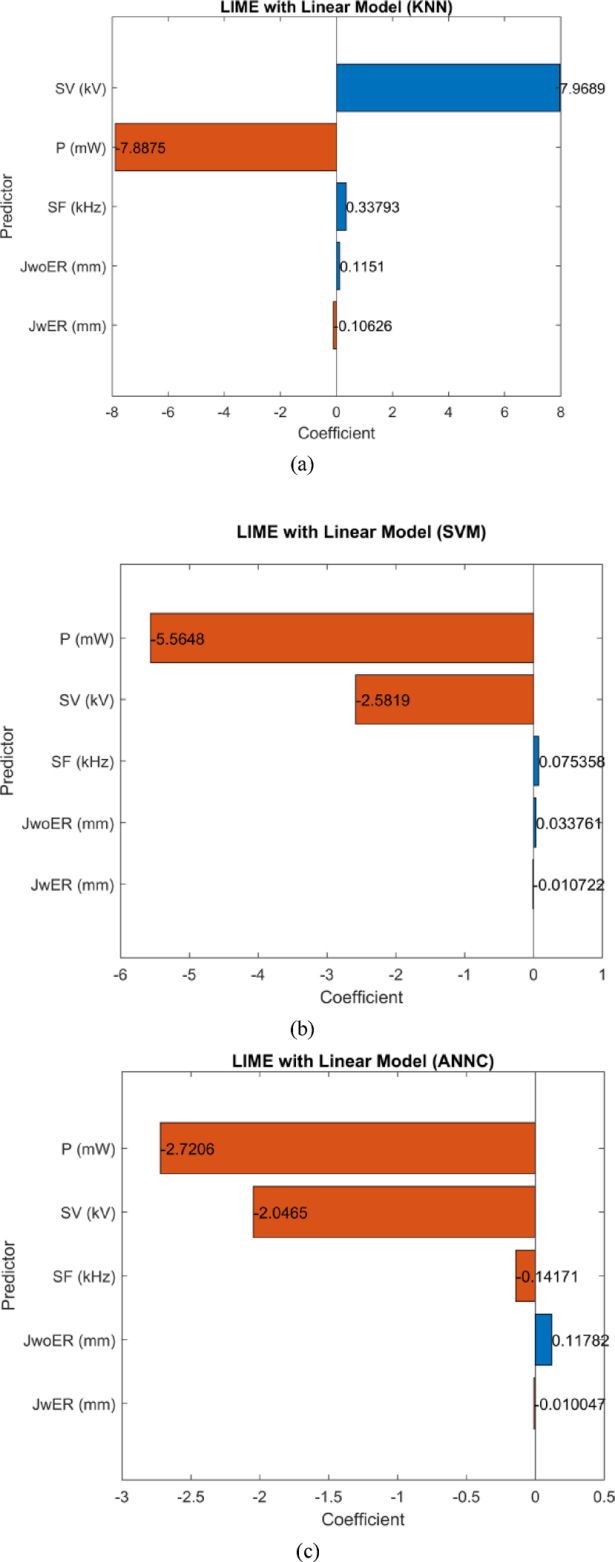
LIME coefficient scores representing relative importance of each parameter on model predictions in: (**a**) KNN, (**b**) SVM and (**c**) ANNC models.

The LIME analysis revealed that all three models considered P and SV as the predominant factors for making predictions on the type of discharge (refer Fig. 15). This is consistent with experimental findings of power consumption being an important indicator on the type of discharge being generated. Typically, higher power consumption indicates transfer of this energy in the form of thermal dissipation leading to the shift from glow to arc discharge. Secondly, voltage level also dictates the thermal dissipation. As observed from the experimental results (refer Table 1), at higher supply voltages there is a tendency to transition to arc discharge. Notably, both SVM and ANNC models showed similar qualitative contributions by the process parameters (refer Fig. 15b,c), whereas KNN showed a distinct sensitivity towards the parameters (refer Fig. 15 a) The negative coefficient scores for P and SV exhibited by SVM and ANNC are realistic due to the fact that higher values of P and SV increase the chances of arc discharge. This verifies the physical interpretation of the SVM and ANNC models.

While the current models, particularly SVM, show excellent predictive capability (especially for stable glow discharge states), we note two limitations: (1) Transition state prediction remains challenging (max 75% accuracy), and (2) Model confidence intervals, while generally narrow (± 1–3% for SVM), could be further improved with larger datasets. Future work will focus on expanding the training dataset and implementing ensemble methods to enhance prediction reliability for transitional states.

### Combinatorial responses effects

**Fig. 16:**
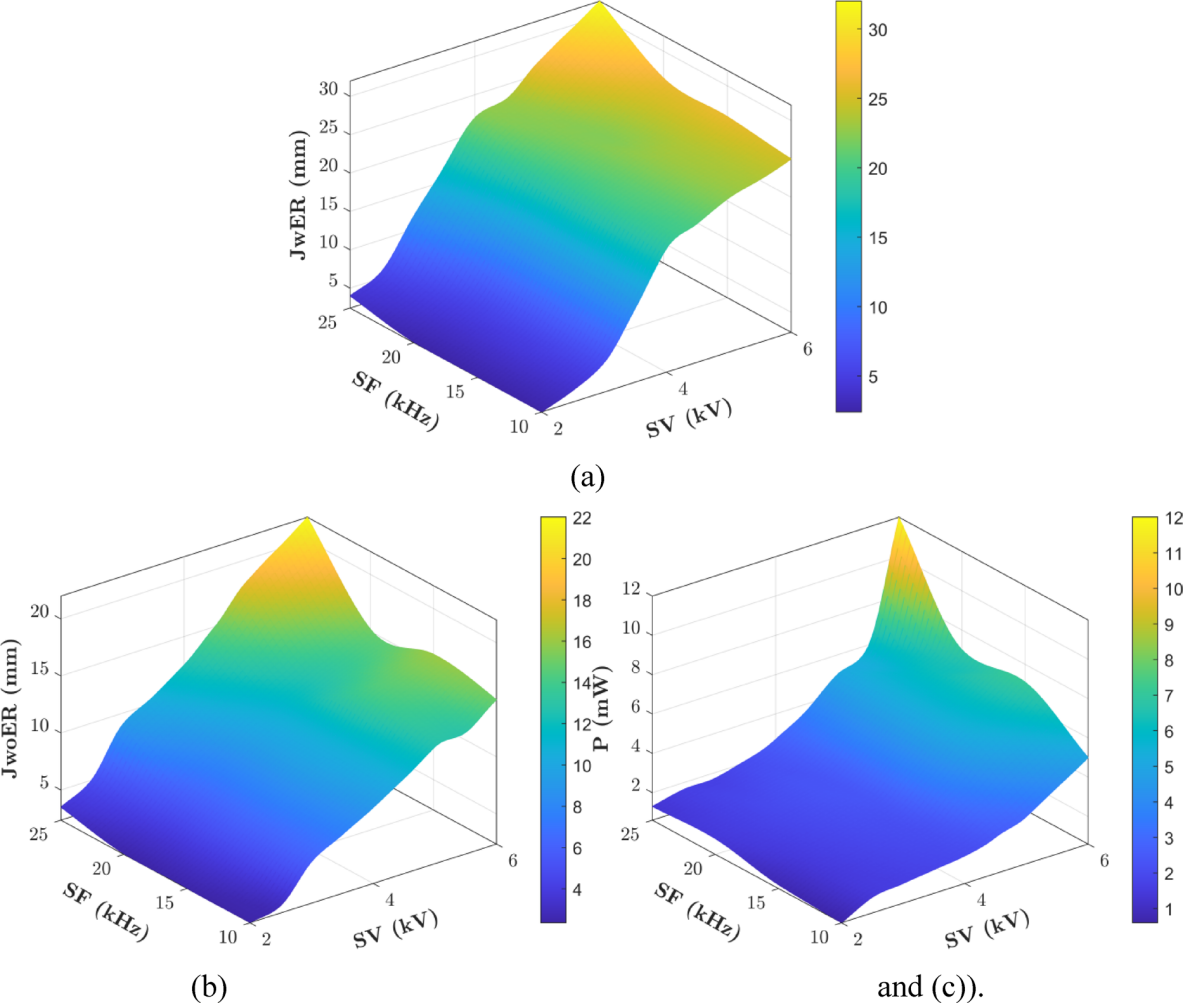
3D surface plots of experimental data shown in Table 1 for (**a**) JwER, (**b**) JwoER, (**c**) P with respect to SV and SF.

The 3D surface plots of the experimental responses, JwER, JwoER, and P were plotted with respect to SV and SF (Fig. 16) to assess their inter-relationships. JwER reaches a maximum at the maximum experimental levels of both SV and SF (6 kV, 25 kHz) (Fig. 16a). JwoER also achieves its maximum value at the maximum experimental settings of both control parameters (6 kV, 25 kHz) (refer to Fig. 16b). However, the effect on P shows an abrupt jump at SV values beyond 5.5 kV. This sudden change also coincides with the transition from glow to arc discharge, clearly demonstrating the relationship between higher power consumption and the transition to arc discharge. By re-emphasizing the sensitivity analysis results, all three responses significantly increased with changes in SV compared to SF.

### Potential of developed device for biomedical applications

Most of the cold atmospheric pressure plasma jets consume power in the range of Watts^14,21^ but our developed setup consumes it in the range of milliWatts with longer jet lengths which is a significant advancement in the technology for building portable plasma devices which are energy efficient. This system generates cold plasma using Argon gas at atmospheric pressure with longer jet lengths (Jet length with end ring) (49 mm) and lower power consumption (43.20 mW) at 9.5 kV/25 kHz, which could potentially be applied for a range of biomedical applications, such as wound healing and sterilization, and surface performance enhancements of bioimplants^3,4^. More detailed discussion on practical application of cold plasma for biomedical application can be found in^46^. Here, it is imperative to note that, this will require in-vitro and in-vivo biomedical laboratory testing and study of biomedical changes and sterilizing effects before its clinical implementation^47^.

### Limitations

The efficacy and safety of cold plasma treatments are significantly influenced by the exposure time and dose. For instance, in cancer therapy, treatment times ranging from 30 s to 5 min have been employed, with variations depending on the cell line and plasma device used^48^. Longer exposure times can enhance the generation of reactive oxygen and nitrogen species (RONS), which are crucial for therapeutic effects but may also increase the risk of cytotoxicity if not properly controlled^49^. Literature indicates that for biomedical applications, the electron temperature (Tₑ) of the plasma should be carefully controlled. Typical electron temperatures in cold atmospheric pressure plasmas range from 1 to 10 eV, corresponding to approximately 11,600 to 116,000 K^50^. Operating within this range is critical to prevent potential damage to biological tissues. Therefore, biological testing (e.g., cell viability, sterilization assays, or tissue interaction studies) are essential before any biomedical use of the cold plasma device can be confirmed.

To overcome these limitations, in future work, we aim to conduct optical emission spectroscopy (OES) to analyze reactive species and measure electron temperature, threshold mapping of the electrical input parameters that induce discharge transitions, and sterility testing and biological assays to correlate plasma states with functional outcomes.

As shown in Table 1, the decrease in jet length beyond a certain voltage threshold is a known phenomenon in atmospheric pressure plasma jets. At higher supply voltages, especially beyond the optimal operation point, increased energy input can lead to thermal instabilities or partial transition toward arc-like behavior. This can cause jet contraction or irregularity rather than elongation, despite increased power consumption. At elevated voltages and frequencies, the plasma may reach a saturation point in ionization efficiency, where further energy input does not proportionally enhance plasma propagation. Additionally, energy losses due to gas heating or increased electron-neutral collisions can reduce the effective jet length. While this behavior is observed and reported, a detailed investigation of the underlying physical mechanisms is an important avenue of future investigation, particularly involving time-resolved imaging and optical diagnostics.

We acknowledge that additional data points, especially beyond the glow-to-arc transition region, would be needed for a more comprehensive understanding and generalization of the model. Future work will focus on exploring the plasma behavior at higher voltages, which will provide a larger dataset that includes the arc discharge regime and allow us to refine and extend the model’s predictive capability. A dedicated follow-up study will be planned to systematically characterize the 5.5-7 kV transition zone with higher sampling density (≥ 50 additional points). This will enable development of a hybrid physics-ML model specifically for transition prediction, combining: (1) stochastic modeling of electron avalanches, and (2) ANN ensembles with uncertainty quantification.

It is important to emphasize here that the scope of the present study was intentionally limited to the *cold plasma regime*, as defined by non-equilibrium discharge characteristics at atmospheric pressure with low gas temperatures. All modeling, optimization, and classification efforts were conducted within this domain to ensure relevance to biomedical applications and avoid confounding effects from arc-like or thermal plasma behavior. While extension into the glow-to-arc transition regime is a valuable direction for future research, the present focus ensures methodological consistency, safety in experimentation, and practical relevance for cold plasma technologies. This study lays the groundwork for broader modeling in future, multi-regime investigations.

## Conclusions

This study presented the results of a coupled machine learning and statistical approach for the modeling and optimization of a floating helix electrode-based DBD-APCPJ system. An FFBP ANN model was developed to predict the APCPJ performance with reasonable accuracy (R^2^ > 0.92) while using a minimal number of neurons to avoid overfitting. The robustness of the model was supported by its alignment with the underlying physics of cold plasma generation.

Importantly, the study focused exclusively on the cold atmospheric pressure plasma (CAP) regime, defined by low-temperature ionized gases in which the electrons are energetic while the bulk gas remains near room temperature. All experimental data and machine learning models—including both the regression and classification models—were developed and validated strictly within this cold plasma operating range (SV ≤ 6 kV). The multi-objective optimization using the desirability function approach yielded optimal input conditions (SV = 5.5 kV, SF = 25 kHz), corresponding to JwER = 29.25 mm, JwoER = 20 mm, and *P* = 5.90 mW.

For discharge zone classification, the Support Vector Machine (SVM) model achieved the highest accuracy (AUC = 0.99) among the three models tested (ANN classifier, KNN, and SVM), again within the cold plasma regime. These results collectively demonstrate the potential of machine learning for modeling and optimizing DBD-APCPJ devices under well-defined operating conditions.

Nevertheless, the findings should be interpreted within the limitations of the present study, especially the constrained dataset and the absence of arc or thermal plasma data. Broader generalization would require expanded experimentation across wider voltage and discharge regimes. The current system may hold potential for biomedical applications, contingent upon further validation through biological testing.

## Data Availability

The data that support the findings of this study are available from the corresponding author, SKB, upon reasonable request.
